# Supplementary material for: The Relationship between Resistance Training Frequency and Muscle Quality in Adolescents
Source: Int J Environ Res Public Health. 2022 Jul 1;19(13):8099. doi: 10.3390/ijerph19138099 (PMC9265918; doi:10.3390/ijerph19138099)
Supplement: Supplementary file 1 [file ijerph-19-08099-s001.zip › ijerph-1744774-supplementary.pdf]

Supplementary Table S1. Association between resistance training (RT: 1 day/week, 2 day/week, 3 day/week, 4 day/week, and 5–7 day/week) and muscle quality among those aged 12–15.

	**P-value of association between RT and MQ**									
	Very Low (1 day/week) vs. Low (2 day/week)	Very Low (1 day/week) vs. Moderate (3 day/week)	Very Low (1 day/week) vs. High (4 day/week)	Very Low (1 day/week) vs. Very High (5-7 day/week)	Low (2 day/week) vs. Moderate (3 day/week)	Low (2 day/week) vs. High (4 day/week)	Low (2 day/week) vs. Very High (5-7 day/week)	Moderate (3 day/week) vs. High (4 day/week)	Moderate (3 day/week) vs. Very High (5-7 day/week)	High (4 days/wk) vs. Very High (5-7 day/week)
	P-value	P-value	P-value	P-value	P-value	P-value	P-value	P-value	P-value	P-value
Model 1	0.6177	0.7561	0.8806	0.9606	0.7264	0.6183	0.3642	0.8407	0.6507	0.8469
Model 2	0.6431	0.7628	0.1935	0.4514	0.7686	0.3945	0.7921	0.2769	0.5321	0.5538
Model 3	0.7192	0.9149	0.2819	0.5323	0.6787	0.4432	0.7285	0.3017	0.4752	0.6234
